# Silicone Substrate with Collagen and Carbon Nanotubes Exposed to Pulsed Current for MSC Osteodifferentiation

**DOI:** 10.1155/2017/3684812

**Published:** 2017-08-23

**Authors:** Daniyal Jamal, Roche C. de Guzman

**Affiliations:** Bioengineering Program, Department of Engineering, Hofstra University, Hempstead, NY 11549, USA

## Abstract

Autologous human adipose tissue-derived mesenchymal stem cells (MSCs) have the potential for clinical translation through their induction into osteoblasts for regeneration. Bone healing can be driven by biophysical stimulation using electricity for activating quiescent adult stem cells. It is hypothesized that application of electric current will enhance their osteogenic differentiation, and addition of conductive carbon nanotubes (CNTs) to the cell substrate will provide increased efficiency in current transmission. Cultured MSCs were seeded and grown onto fabricated silicone-based composites containing collagen and CNT fibers. Chemical inducers, namely, glycerol phosphate, dexamethasone, and vitamin C, were then added to the medium, and pulsatile submilliampere electrical currents (about half mA for 5 cycles at 4 mHz, twice a week) were applied for two weeks. Calcium deposition indicative of MSC differentiation and osteoblastic activity was quantified through Alizarin Red S and spectroscopy. It was found that pulsed current significantly increased osteodifferentiation on silicone-collagen films without CNTs. Under no external current, the presence of 10% (m/m) CNTs led to a significant and almost triple upregulation of calcium deposition. Both CNTs and current parameters did not appear to be synergistic. These conditions of enhanced osteoblastic activities may further be explored ultimately towards future therapeutic use of MSCs.

## 1. Introduction

Stem cells have the potential to revolutionize contemporary medicine and therapy methods. They enable us to screen new drugs, investigate the causes of birth defects, and understand the development of complex organisms from a single cell [1, 2]. More importantly, their greatest potential lies in cell-based and tissue engineering therapies to combat a range of diseases [3]. The idea of growing functional tissues and organs is no longer farfetched. Hematopoietic stem cells from bone marrow are one of the few clinically approved stem cells which are proven to increase the survival rate for thousands of patients [4]. Bone marrow-derived mesenchymal stem cells, or the blood stem cells that are nonhematopoietic, are currently being used in clinical trials and show promise in improving recovery based on early indicators [5]. Mesenchymal stem cells (MSCs) are multipotent stromal cells in connective tissues capable of differentiating into a variety of specialized and functional cells, including osteoblasts, chondroblasts, myocytes, and adipocytes. MSCs are easy to isolate, propagate to sufficient numbers, can be differentiated using simple chemicals, and have been proven safe [6]. They can be utilized as an autologous graft (from the same individual) or an allograft (from another human source) which, when introduced into the patient, leads to less likelihood of immune rejection, thus making them ideal for transplants and in vivo applications [7, 8]. Gaining control of MSCs differentiation pathways in vitro and within their local environment is a crucial feat for an efficient clinical approach. For autologous human adipose tissue-derived MSCs, enhanced differentiation into osteoblastic phenotype can be advantageous and may be used with matrix biomaterials for regeneration of the patient's own damaged bones due to fractures, osteoporosis, and deformities. The development of new technologies such as this tissue engineering approach is very important since millions of people [9] suffer from bone injuries on a yearly basis. A possible differentiation enhancer is the application of an external stimulus to stem cells in the form of electricity [10–12].

Bones contain a matrix of collagen proteins, which are piezoelectric materials that accumulate small electrical charges when subjected to mechanical stresses, leading to stimulation and deposition of intracellular calcium during fracture healing [13]. Biophysical stimulation methods [9] such as pulsed electric currents and electromagnetic fields have been clinically shown to significantly improve bone fracture repair—even the healing of nonunions, which is an advanced stage of fracture where healing is ceased by the body [14–16]. Rat tibial osteoporosis was reversed back to nonosteoporotic condition through capacitively coupled electric currents [17]. These definitive bone healing activities imply that quiescent local MSCs are activated for osteogenesis directly or through indirect effects of biological and chemical processes influenced by bursts of electricity [18] with varying strengths and frequencies. To provide a more efficient medium of electrical conduction, covalently linked elemental carbon in the form of carbon nanotubes (CNTs) can be introduced into the stem cell-adhesion substrates. CNTs are hexagonally ordered carbon atoms that form a lattice in the shape of hollow cylindrical tubes [19]. A single tube's outer diameter typically ranges from 10 nm to 50 nm. CNTs are primarily available in two forms: single-walled and multiwalled nanotubes, which vary in conductivity, fiber strength, and applications. They have exceptional mechanical properties, reported to be one of the strongest materials to be discovered. CNTs can also be chemically modified allowing for the attachment of functional carboxyl, amine, and hydroxyl groups within their structure for further functionalization. They are highly electroconductive; hence, they can enhance the flow of electric current.

Accordingly, in this study, the osteogenic differentiation effects of low intensity pulsed electric currents on cultured and induced MSCs using chemical factors were tested. Additionally, the presence of electrically conductive carbon nanotubes in cell-attachment silicone-collagen composite films was investigated for their MSC differentiation ability.

## 2. Materials and Methods

### 2.1. Materials

**Figure 1:**
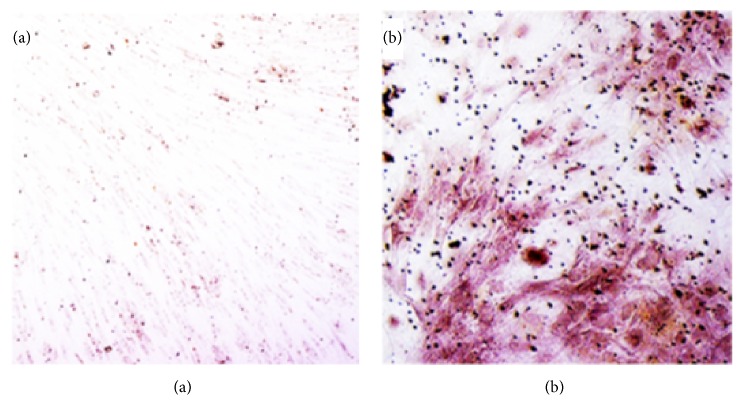
MSCs grown on plasma-treated polystyrene surface (a) left uninduced and (b) induced with osteogenic culture medium and then stained with Alizarin Red S. Induced cultures showed more rounded morphological features and a 21-fold increase in red dye absorption.

Sylgard® 184 Silicone Elastomer Kit was purchased from Dow Corning (Greensboro, NC) for silicone matrix polymerization. Composite discontinuous phase components: type I collagen was prepared in the lab from rat tails generously given by Dr. Mark Van Dyke's lab (Virginia Tech, Blacksburg, VA, from IACUC-approved animal protocol), while carboxylic acid- (COOH-) functionalized graphitized multiwalled carbon nanotubes (CNTs with outside diameter = 20 to 30 nm and length = 10 to 30 *μ*m) were obtained from Cheap Tubes (Cambridgeport, VT). Common lab reagents as well as N-(3-dimethylaminopropyl)-N′-ethylcarbodiimide hydrochloride (EDAC), N-hydroxysulfosuccinimide sodium salt (sulfo-NHS), MES buffer, poly(dimethylsiloxane-co-(3-aminopropyl)methylsiloxane), poly(dimethylsiloxane), bis(3-aminopropyl) terminated, 10% neutral-buffed formalin (NBF), Alizarin Red S, and dimethyl sulfoxide (DMSO) were purchased from Sigma-Aldrich (St. Louis, MO). Deionized water was used as a solvent, unless stated otherwise. Human adipose tissue-derived mesenchymal stem cells (MSCs) were acquired from ATCC ([PCS-500-011] Manassas, VA). These cells were reported by ATCC to be positive (90% to 100%) for MSC markers, CD29, CD44, CD73, CD90, CD105, and CD166, and negative (<3%) for surface markers (non-MSCs like hematopoietic cells, blood cells, and endothelial cells), CD14, CD31, CD34, and CD45. ATCC, as well as our lab, has tested their in vitro differentiation into adipocytes and osteoblast (Figure 1) on tissue culture-treated plates and on scaffolds with collagen. Maintenance culture medium (CM) for these primary cells utilized Dulbecco's Modified Eagle's medium (DMEM) high glucose (4.5 mg/L), 10% fetal bovine serum, and Gibco® Antibiotic-Antimycotic Reagent (Thermo Fisher Scientific, Waltham, MA). Osteogenic culture medium (OM) for their differentiation into osteoblast-like cells contained CM with 10 mM *β*-glycerol phosphate, 100 nM dexamethasone, and 200 *μ*M ascorbic acid 2-phosphate.

### 2.2. Collagen Extraction

Type I collagen protein fibers were isolated from rat tail tendons based on modified Bornstein's methods [20]. Briefly, tendon bundles were dissected out, dissolved in acetic acid solution, precipitated with high salt, and centrifuged, and precipitates containing collagen were redissolved in acid. The solutions were then dialyzed for solvent exchange into dilute hydrochloric acid, freeze-dried, and stored at 4°C. The collagen yield was determined to be 175 mg per tail. A 1% (10 mg/mL ≈ 10 mg/g) collagen solution was made by dissolving the cotton-like powder in 1 M HCl.

### 2.3. Composite Film Preparation

Substrates composed of polydimethylsiloxane (PDMS; silicone) continuous phase with collagen and CNTs discontinuous phases were fabricated as follows. Sylgard base and curing reagents, collagen solution (1% in 1 M HCl), and CNTs were weighed, according to the values in Table 1, into wells of 6-well tissue culture-treated plates (Thermo Fisher Scientific) and then slowly mixed to make a homogenous solution. Samples were placed overnight in a vacuum oven at 70°C to remove air bubbles and to cure the film substrates. Three groups of composites, namely, high CNTs with ~10% (m/m) CNTs, low CNTs with ~5% CNTs, and no CNTs (negative control; without CNTs) were made.

Sylgard reagents were also mixed with different ratios of amine-linked PDMS: poly(dimethylsiloxane-co-(3-aminopropyl)methylsiloxane) and poly(dimethylsiloxane), bis(3-aminopropyl) terminated prior to curing to potentially introduce amine groups to the silicone matrix.

**Figure 2:**
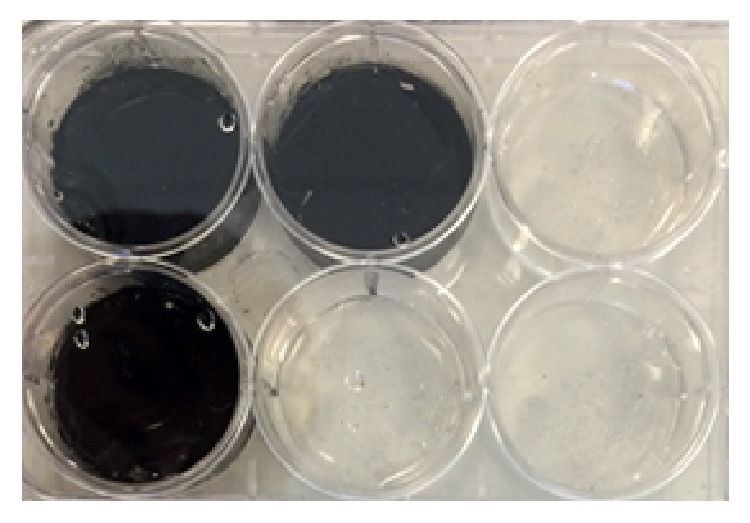
Plates containing surface-crosslinked collagen on films with no CNTs (clear) and high CNTs (black).

To ensure surface adhesion of cells, a collagen coating was applied onto the silicone-based substrates. Collagen solution (60 *μ*M in 1 mM HCl) was mixed with a carbodiimide crosslinker EDAC (150 *μ*M) and sulfo-NHS (75 nM) in MES buffer, pH 6.0, for 15 min at room temperature (RT) to activate collagen. A 1.5 mL aliquot of this solution was added to the surface of the cured films and reacted for 3 hours at RT. Complete reaction expected a yield of 36 mg of collagen (for MW ~ 400 kDa) or a surface area density of 3.8 mg/cm^2^ for an area of 9.5 cm^2^. Collagen was expected to crosslink to the carboxylic acid groups of the functionalized CNTs and the collagen present within the material. After the reaction, films were washed with phosphate-buffed saline (PBS) thrice to remove uncrosslinked reactants and products (Figure 2).

### 2.4. Measurement of Electrical Properties

**Figure 3:**
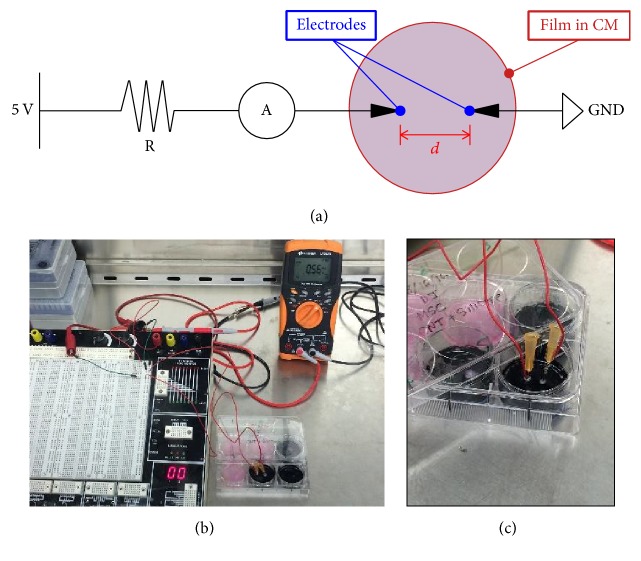
(a) Circuit diagram and (b) actual experimental setup for application of electric current to the composite films soaked in culture medium. (c) Close-up image of the electrodes penetrating the silicone substrate with collagen and carbon nanotube fibers. *R* = resistor, *A* = ammeter, GND = ground, and *d* = distance separating the two electrodes.

Film substrate groups (high CNTs, low CNTs, and no CNTs) in wells of 6-well plates were washed thrice and then soaked in 1.5 mL of CM. Positive and negative electrodes were immersed into the liquid culture medium and penetrating the substrates at 2 cm apart (*d* = 0.02 m). An electrical circuit (Figure 3(a)) was constructed connecting the electrodes and powered by a 5 V source with a 220 *Ω* resistor on a PB-505 circuit design trainer (Global Specialties, Yorba Linda, CA). A multimeter (ammeter function) was used to monitor the direct electrical current (*I*). Current values were measured at 1 min intervals for 10 min. Electrical conductivity (*σ*) was computed based on the equation

(1) $$\begin{array}{c} \sigma =\frac{I}{Vd}, \end{array}$$

where *I* is average stable electric current in A, *V* is electric potential in V, and *d* is distance between electrodes in m.

### 2.5. Mesenchymal Stem Cell Culture on Silicone-Based Films

MSCs were revived, proliferated, and cultured up to sufficient numbers in T-75 tissue culture flasks (Thermo Fisher Scientific) with culture medium, CM, at 5% CO_2_, > 90% relative humidity, and 37°C (in a mammalian cell incubator). CM was replaced twice a week. Trypsinized and CM-resuspended MSCs were seeded at 9 × 10^3^ cells/well onto the test substrates: high CNTs and no CNTs then incubated for 24 hours at 37°C. The next day, nonadherent cells (cells in suspension) were counted using a hemocytometer. Cell attachment was reported as

(2) cell  attachment=initial  cell  count−cells  in  suspensioninitial  cell  count.

Adherent cells growing on films were allowed to proliferate for 7 days in CM.

### 2.6. Osteogenic Differentiation with Pulsed Current

The culture medium of proliferating MSCs on composite substrates was replaced with osteogenic medium, OM, for 2 weeks to induce differentiation of mesenchymal stem cells into the osteoblastic phenotype. For each of the two groups, high CNTs and no CNTs, two subgroups were created: with current (experimental) and no current (control). For the “with current group,” after 3 days in OM, a total of 6 wells were subjected to pulsed electrical currents (Figures 3(b)-3(c)), 3 for high CNTs and 3 for no CNTs. Low milliampere-level currents, 0.5 to 0.6 mA (at 5 to 6 mS/m conductivity), were applied for a 2 min duration followed by a 2 min duration of no current for a total of 20 minutes (5 cycles at 240 s^−1^ ~ 4 mHz frequency). Ohm's Law was utilized to calculate the total circuit resistance, which included the resistor, liquid medium, and composite film resistance. The total resistance was in the range of 8.3 to 10 k*Ω*. Pulsed current application was performed in a Biosafety Level 2 (BSL-2) cabinet twice a week prior to the culture medium change.

At the 2-week endpoint after OM induction, media were removed from the samples; 10% NBF was added for 15 min to fix cells. Representative sample regions for the “no current” groups were saved for elemental analysis. The remaining samples were then washed thrice with PBS and twice with water and stained with 250 *μ*L per well of 2% Alizarin Red S, pH 4.2, for 5 min for deposited calcium staining. The stained composite films with MSC cells were destained with water thrice and solubilized with 750 *μ*L DMSO per well for 10 min. Aliquots (200 *μ*L) of liquid samples were transferred into wells of a 96-well plate and their absorbances were read in an iMark™ spectrometer (Bio-Rad, Hercules, CA) at a wavelength of 490 nm. DMSO solvent was used as a blank. Colorimetric readings were normalized on sample surface areas. Films before and after DMSO treatment looked similar, indicating that DMSO only dissolved cells and Alizarin Red S-calcium deposits, making the colorimetric test reliable for calcium precipitate detection.

### 2.7. Elemental Analysis of Osteogenic-Induced Cells

Sampled spots from the no current groups (high CNTs and no CNTs) were washed in water, air-dried, and then loaded into the sample holder of Q250 SEM scanning electron microscope (FEI, Hillsboro, OR) with UltraDry™ (energy-dispersive X-ray spectroscopy) EDS detector (Oxford Instruments, Oxfordshire, England, UK). SEM was operated at 30 kV and 1 mm^3^ areas were selected for EDS analysis. Elemental carbon (C) and calcium (Ca) mass fractions were obtained and Ca/C ratios were reported.

### 2.8. Statistical Analysis of Data

All experiments were performed with sample sizes (replicates) of *n* ≥ 3. Computed values and graph points were reported as average ±1 standard deviation. Excel (Microsoft, Redmond, WA) and Prism (GraphPad, San Diego, CA) software were used for generating bar graphs and scatter plots. Student's *t*-test and one-way analysis of variance (ANOVA) with Tukey's post hoc multiple comparison analyses were made with Prism at 95% confidence intervals and 5% probability of type I error.

## 3. Results and Discussion

### 3.1. Fabricated Composite Substrates

**Figure 4:**
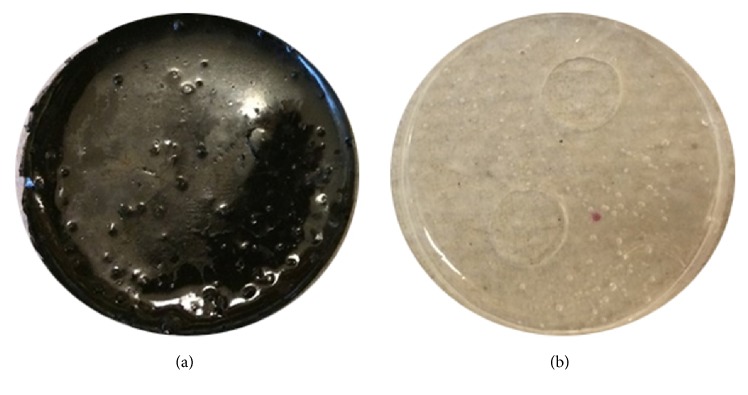
Fabricated silicone-based substrates with high CNTs (a) and no CNTs (b).

Silicone-based composite films were synthesized (Figure 4) containing surface-crosslinked collagen for cell attachment and CNTs for promotion of increased electrical conductivity. The elemental carbon tubes were evenly dispersed creating a uniform black layer. Multiple washes of PBS demonstrated stability of the CNTs within the films since no loose particles were detected from the waste solution. Immobilization and crosslinking into the silicone matrix with collagen thereby prevented the possible cell internalization and cytotoxicity of free fibers of CNTs in suspension [21].

In groups with no CNTs, addition of 0.03% (m/m) = 0.3 mg/g collagen (Table 1) and surface collagen coating did not affect the visible appearance and transparency of the films relative to PDMS films alone. The amine-linked PDMS additives tested, poly(dimethylsiloxane-co-(3-aminopropyl)methylsiloxane), and poly(dimethylsiloxane), bis(3-aminopropyl) terminated, were not used as matrix precursors since they did not allow for the polymerization and complete curing of the silicone-based films.

### 3.2. Conductivity of Films in Culture Medium

**Figure 5:**
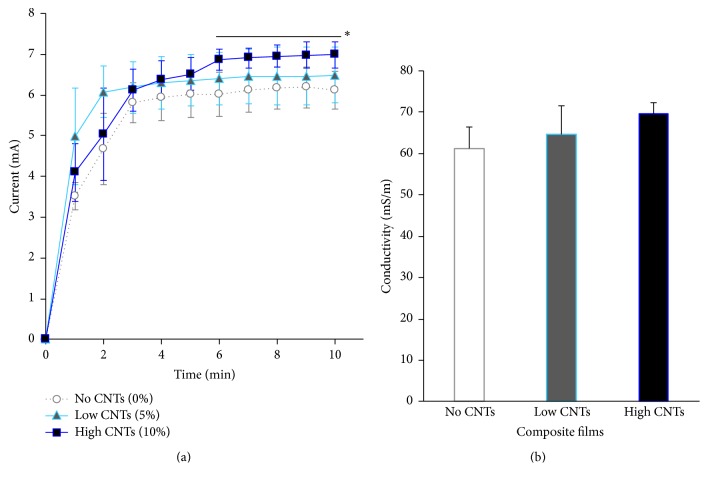
(a) Measured direct electrical currents (in mA) at constant voltage in composite films (no CNTs, low CNTs, and high CNTs groups) immersed in culture medium. ^*∗*^*p* < 0.05 for high CNTs versus low CNTs and no CNTs at currents from 6 to 10 min time points (horizontal line). (b) Electrical conductivity (in mS/m) derived from 1 per system resistance and electrodes distance.

Electrical current (direct current, DC) passing between electrodes placed 2 cm apart along a film, immersed in culture medium at 5 V electric potential connected to a 220 *Ω* resistor, was found to increase from 0 to about 7 mA over the 10 min time period (Figure 5(a)). At the 6 to 10 min marks, the current flattened indicating stability. ANOVA and Tukey's test showed that the mean values of the current transmission of films with 10% CNTs (high CNTs) were significantly greater (*p* < 0.05) than those with 5% CNTs (low CNTs) and substrates without CNTs (no CNTs). Low CNTs generated significantly higher DC current (*p* < 0.05) than those of negative control films. After stabilization, the high CNTs group allowed 11.1% more electrical current than the no CNTs group and 5.1% more than the low CNTs group. The total system resistance from the resistor and substrate and liquid medium resistance were reported as 827 *Ω* (no CNTs), 782 *Ω* (low CNTs), and 760 *Ω* (high CNTs). Accordingly, the corresponding electrical conductivities (Figure 5(b)), computed in mS/m at *d* = 0.02 m, were determined to be highest (although not statistically significant) in films with high CNTs (at 69.4 mS/m) compared to the other two groups (low CNTs and no CNTs). The conductivity values obtained were close to that of drinking water and within the expected range of silicone polymers [22, 23]. Increasing amounts of CNTs led to increased conductivity of the composite silicone-based material; hence, the 10% CNT formulation was utilized in subsequent cell culture and induction experiments for potentially higher electrical responses.

### 3.3. Adhesion of Mesenchymal Stem Cells onto the Substrates

**Figure 6:**
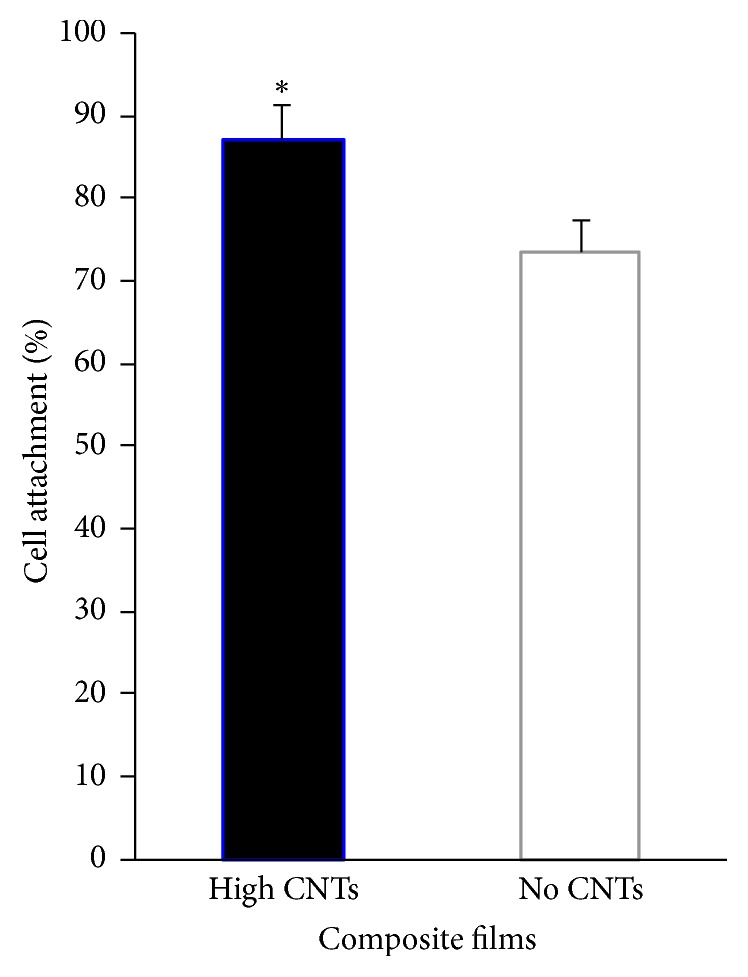
Adhesion of mesenchymal stem cells on silicone-collagen substrates with high CNTs and no CNTs. ^*∗*^*p* < 0.05 for high CNTs versus no CNTs.

Seeding MSCs on composite films after 24-hour incubation resulted in significantly more attached cells (*p* < 0.05) on the silicone-collagen composites with 10% CNTs (high CNTs) compared to those without (0% CNTs; no CNTs) (Figure 6). The presence of CNTs led to 13% more initial cell adhesion on the film surface (87% on high CNTs versus 74% on no CNTs). This result implies that carbon nanotube fibers aid in cell attachment, possibly due to the presence of charged functional carboxylic acid groups promoting extracellular matrix adsorption, which in turn allows cell binding through their integrin receptors. MSCs are adhesion-dependent cells and can only survive and differentiate after successful substrate attachment. Collagen added as coatings (≤36 mg) and mixed in bulk films (~0.3 mg) successfully provided the initial substrate adhesion of these cells. A previous study showed similar enhancement of stem cell adhesion due to reinforcing CNTs within the substrate material [24].

### 3.4. Pulsed Current Exposure during Cell Differentiation

**Figure 7:**
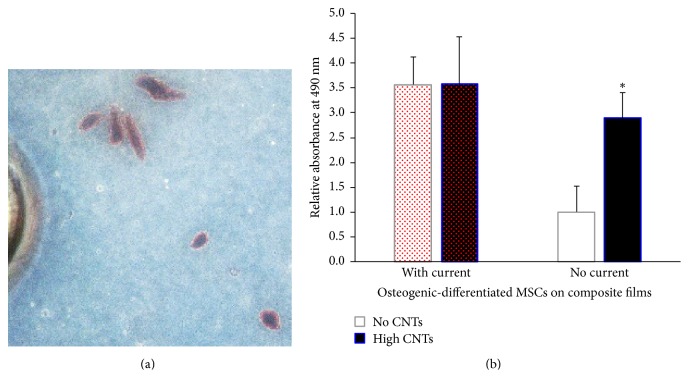
(a) A representative image of Alizarin Red S-stained cell clusters on the surface of no CNTs silicone-collagen film. (b) Absorbance spectroscopy at 490 nm (red intensity) of DMSO-solubilized Alizarin Red S-calcium crystals on composites with high CNTs and no CNTs, with applied electrical stimulation (with current) and without it (no current control). ^*∗*^*p* < 0.05 for high CNTs versus no CNTs at a no current condition.

**Figure 8:**
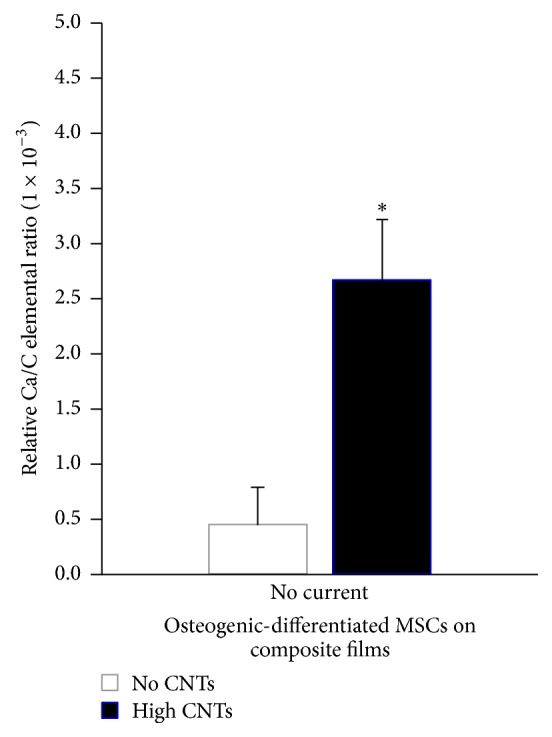
Relative amounts of Ca to C on induced MSCs on silicone-collagen substrates with high CNTs and no CNTs. ^*∗*^*p* < 0.05 for high CNTs versus no CNTs.

The electrical current applied to MSCs on films was in the submilliampere level, specifically in the range of 0.5 to 0.6 mA, and delivered at interrupted regular intervals (i.e., noncontinuous and pulsatile). These exposure conditions enable current to flow through cells without killing them [15, 25]. After the 2-week time point, where MSCs were subjected to induction by chemical factors, namely, glycerol phosphate, dexamethasone, and ascorbic acid (vitamin C), as well as with pulsed electrical current, cells on the surface of translucent no CNTs substrates showed dark red staining with Alizarin Red S (Figure 7(a)), indicating calcium deposition and osteoblastic lineage cell differentiation. Contrastingly, those grown on black films with high CNTs (Figure 4) were not observed using light microscopy because of black-body light absorption. Alizarin Red S-calcium precipitates were quantified by DMSO solubilization and subsequent spectrophotometric analysis at 490 nm (red signal). ANOVA and Tukey's multiple comparison tests showed that the presence of CNTs alone (high CNTs versus no CNTs at no current) led to significantly more production of deposited calcium (*p* < 0.05; 2.9-fold increase) based on Alizarin Red S staining (Figure 7(b) right). Furthermore, elemental analysis of film surfaces demonstrated significantly elevated (*p* = 0.0044), 6-fold calcium relative to carbon level increase (Figure 8). These results indicated that carbon nanotubes induced more osteoblast differentiation, which may primarily be due to more cells adhering onto the high CNTs composites (Figure 6), leading to high survival of cells and increased osteogenic response.

The application of pulsed current in silicone-collagen groups only (with current versus no current at no CNTs) led to significantly more Alizarin Red S staining (*p* < 0.01), suggesting that pulsed current was beneficial towards induction of more MSCs, directly by stimulating few cells to enable calcium deposition or indirectly by initially enabling few cells to proliferate prior to differentiation. The higher the cell number, the greater the relative amount of calcium phosphate that precipitated. Other researchers demonstrated similar enhanced differentiation into osteoblast-like cells after biophysical electrical stimulation, like pulsed electromagnetic field and alternating currents [15, 26]. Conflicting studies with negative impacts in stem cell growth and differentiation [27, 28], however, suggest the need for current delivery with optimal properties including the right frequency, magnitude, and duration of cell exposure; otherwise, unwanted effects are observed.

Groups containing high CNTs (with current versus no current at high CNTs) showed elevated response with application of pulsatile current (1.8-fold increase), although nonsignificant (*p* > 0.05; Figure 7(b) black bars). This could indicate that longer duration of current exposure or longer culture time may be needed to observe a significant effect of pulsed current in composite films with 100 mg/g of carbon tubes. Alternatively, the amount of cells on composite films may have reached saturation; hence, any increased response was not sensitive for detection. Further testing is recommended.

The current film substrate composition containing carbon nanotubes (high CNTs) can be utilized as an in vitro preconditioning culture system for production of osteoblasts that can ultimately be transplanted into hosts for bone regeneration therapy. Since free CNTs are cytotoxic [21] at high concentrations in the body, they can be used as a substrate additive just for cell culture but not for direct implantation into patients.

Pulsed current did not affect the osteogenic calcium activity of differentiated MSCs on silicone composite films (high CNTs versus no CNTs at with current group). The high CNTs and no CNTs groups demonstrated similar levels of absorbance (*p* > 0.05; Figure 7(b) left). Submilliampere-level electric currents were able to transmit across the media containing the composite films, cells, and liquid culture medium; thus, the presence of CNTs did not matter. The average relative absorbance of groups with current was found to be 3.572 ± 0.002, compared to the 1.942 ± 1.332 value for no current. Application of electric current led to 1.8 times higher osteoblastic calcium deposition activity.

Osteogenic induction based on calcium deposition staining showed relatively high activities on groups with pulsatile current (Figure 7(b) left). To further improve MSCs differentiation, groups with longer exposure to electric current (i.e., more cycles: 10 and 20 cycles; longer differentiation period: 3 weeks and 4 weeks) will be added in the follow-up study. Results from these additional experimental groups will enable us to determine the temporal conditions that generate the greatest osteodifferentiation using the current system.

## 4. Conclusions

In summary, this study showed that silicone (PDMS) composites with cell-adhesive collagen and stable reinforcing CNTs were successfully made into films. The immobilized CNTs provided increased electrical conductivity to the substrate, enabled more MSC attachment, and allowed for increased surface calcium deposition when chemical inducers, namely, glycerol phosphate, dexamethasone, and vitamin C, were added to the cell culture media. Application of pulsatile, low intensity, and low frequency external DC electrical current significantly enhanced osteoblastic response on silicone-collagen films without CNTs. The combined differentiation effects of CNTs and pulsed current were found to be nonsynergistic or nonadditive in the tested system. These silicone-collagen substrates with CNTs may further be developed for enhancement of mesenchymal differentiation into osteoblasts which can be used in the clinic for treatment of patients with bone injuries. Other potential applications of CNTs in bioengineering include nanotechnology, microelectronics, and medical devices. On the other hand, in composites devoid of CNTs, delivery of pulsed electric current can also be employed towards MSC differentiation in vitro or in vivo in combination with piezoelectric, degradable, implantable, and biocompatible scaffolds as tissue-engineered constructs for bone repair and regeneration.

## Figures and Tables

**Table 1:** Composition of experimental silicone-based films.

*C*_*i*_	Components	High CNTs		Low CNTs		No CNTs	
		*m*	*C*_*f*_	*m*	*C*_*f*_	*m*	*C*_*f*_
100%	Sylgard base	0.75	68.81%	0.75	72.82%	0.75	76.53%
100%	Sylgard curing	0.2	18.35%	0.2	19.42%	0.2	20.41%
1%	Collagen	0.03	0.03%	0.03	0.03%	0.03	0.03%
	HCl		0.05%		0.05%		0.05%
	Water		2.68%		2.83%		2.98%
100%	CNTs	0.11	10.09%	0.05	4.85%	0	0%

	*Total*	*1.09*	*100%*	*1.03*	*100%*	*0.98*	*100%*

*m* = mass (g), *C*_*i*_ = initial concentration (m/m), and *C*_*f*_ = final concentration (m/m).
